# Advanced Epoxy Resin/Boron Nitride Composites for High-Performance Electrotechnical Applications and Geological Instrumentation

**DOI:** 10.3390/ma18214860

**Published:** 2025-10-23

**Authors:** Alina Ruxandra Caramitu, Iustina Popescu, Mihai Grigoroscuta, Andrei Kuncser, Paul Constantin Ganea, Andrei Galatanu, Magdalena Galatanu, Gheorghe Aldica, Petre Badica, Mihail Burdusel, Adriana Mariana Borș

**Affiliations:** 1 National Institute for Research and Development in Electrical Engineering ICPE-CA, Splaiul Unirii Street 3131, District 3, 030138 Bucharest, Romania; alina.caramitu@icpe-ca.ro; 2 Geological Institute of Romania-IGR, Caransebes Street 1, District 1, 012271 Bucharest, Romania; 3 National Institute of Materials Physics, Atomistilor Street 405 A, 077125 Magurele, Romania; mihai.grigoroscuta@infim.ro (M.G.); akuncser@yahoo.com (A.K.); paul_ganea@yahoo.com (P.C.G.); gala@infim.ro (A.G.); magdalena.galatanu@infim.ro (M.G.); aldica2000@yahoo.com (G.A.); badica2003@yahoo.com (P.B.); 4 National Research and Development Institute for Optoelectronics INOE 2000, IHP, Cutitul de Argint Street 14, District 4, 040558 Bucharest, Romania; bors.ihp@fluidas.ro

**Keywords:** epoxy resin composites, boron nitride, thermal conductivity, dielectric properties, electrotechnical applications, geological equipment

## Abstract

Composites were obtained by using a commercial bicomponent epoxy resin (containing Al_2_O_3_ and SiO_2_) and cubic BN (cBN) (0–50 wt.%). Two preparation methods were used: (a) the cBN powder was first mixed with the resin base and further with the hardener, and (b) the cBN powder was first mixed with hardener and afterwards with the resin base. Both methods show a similar enhancement trend in the thermal conductivity from 0.77 W/mK in the resin without additive to 1 and 1.5 W/mK in the ones filled with 30 and 50 wt.% cBN, respectively. A reasonable decrease in bending strength from 74 for 0 wt.% cBN to 70 or 64 MPa for 30 wt.% cBN added by method (a) or (b), respectively, occurs. The bending strain at breakage decreased from 5.58% to 2.65 or 3.78%. High sensitivity vs. processing was also found for electrical properties measured for frequencies of 1–10^7^ Hz. An increasing amount of cBN decreased the room temperature conductivity, dielectric constant, and dielectric loss tangent and modified the shape of the curves vs. frequency. However, in samples with 30 wt.% or more of cBN, a partial recovery to a high insulating state was observed.

## 1. Introduction

The rapid development of integrated, miniaturized electronic devices with high power density imposes new demands on current heat dissipation materials [1]. To ensure the lifetime and reliability of electronic devices, the heat generated by electronic components is traditionally transferred to a heat sink via a thermal interface material (TIM) with high thermal conductivity (TC) [2]. Efficient heat dissipation is the critical characteristic of these materials [3,4]. From the perspective of heat conduction mechanisms, heat transfer mainly occurs through three modes: thermal radiation, thermal convection, and thermal conduction. Among these, thermal conduction is the most effective way to achieve efficient heat dissipation, being dependent on lattice vibrations and free electron movement in solid materials [5]. In practice, materials with high TC are also required to fulfil other criteria. They should have a convenient set of other excellent properties such as good adhesiveness, chemical stability, mechanical properties, thermal shock resistance, low shrinkage, excellent dielectric properties, and competitive cost, as well as specific features such as easy processing and good protection against moisture, fire, dust, vibration, and electromagnetic noise. These complex characteristics depend on applications spanning from wearable electronics, thermal interface materials, battery thermal management, dielectric capacitors, electrical and power equipment, solar thermal energy storage, biomedical applications, carbon dioxide capture, radiative cooling [6], and others.

Compared to metallic materials, which have relatively high thermal conductivity, polymer materials offer several advantages such as low weight, electrical insulation, and corrosion resistance. They may also show convenient optical and mechanical properties such as transparency and flexibility [7,8,9]. Polymer materials also reduce the contact resistance of heat sources [6,9]. However, traditional polymers face challenges in meeting the high heat dissipation requirements due to their inherently low thermal conductivity—usually in the range of 0.1−0.5 W/mK [7,10,11]. A higher crystallinity and chain alignment, a lower entangling, and stronger interchain interactions can push thermal conductivity to high values (up to 62 W/mK) as has been shown for polyethylene (PE) films obtained in Ref. [12]. At present, highly conductive polymers face scaling-up challenges because they are small in size and are obtained by complicated preparation processes.

A straightforward solution to achieve efficient heat dissipation is to design and fabricate polymer matrix composites reinforced with thermally conductive fillers [13,14]. The general concept is based on the fundamental percolative nature of the thermal conductivity [4]. This means that the strategies to enhance thermal conductivity of a composite should provide a convenient and continuous path for heat flow involving both the polymer and the filler or fillers. To do so, according to Ma et al. [13], one can concentrate on filler functionalization and processing, filler hybridization and coating (e.g., core-shell fillers), filler orientation and network, and use of multiple fillers. This also indicates that the type, features and compatibility of the polymer and of the fillers are important. It is also noteworthy that criteria to search for materials with extreme thermal conductivity are currently not fully unveiled [5].

Different articles review polymer composites for thermal management [4,5,6,7,9,11,13,15,16,17,18]. There has been remarkable progress in recent years. Among the most studied fillers are the BN materials containing allotropes [19] that can form structures with either sp^2^ or sp^3^ bonds [20]. From bulk to zero-dimensional, the allotropes of BN are cubic BN (cBN), wurtzite BN (w-BN), hexagonal BN (hBN), rhomboedral BN (rBN), single and multiwall BN nano tubes (BNNT), BN nano ribbon (BNNR)/sheet (BNNS) with zigzag (ZBNNR) and armchair edges (ABNNR), BN nano cages, and quantum dots. The BN materials show high thermal conductivities, up to ~2000 W/mK in BNNR, and it is no surprise that many papers focus on BNNR preparation and use in polymer composites with high thermal conductivity for thermal management. To achieve high thermal conductivities in BNNR and BNNT composites, several authors obtained oriented fillers taking advantage of the materials’ anisotropy. This complicates processing and increases the costs. Moreover, the low yield of BN ribbons/sheets, the fact that they are difficult to control and possess many defects, and their relatively small lateral size restricts the large-scale application of BNNS [16].

Remarkably, isotropic cBN also has a relatively high thermal conductivity: up to 1300 W/mK. For comparison, bulk h-BN has a thermal conductivity as high as ~390 W/mK (for in (ab)-plane bulk h-BN versus about half of the indicated value for the out of plane along *c*-axis thermal conductivity). Both hBN and cBN are excellent low-density (2.27 g/cm^3^ for hBN and 3.48 g/cm^3^ for cBN) electrical insulators. While hBN is soft and lubricious, cBN is hard and brittle. Hexagonal BN is a popular filler in polymer TC composites [13,21,22,23,24,25,26,27,28,29,30,31,32,33]. Although cBN is recognized as a useful filler in Al–matrix composites, promoting TC improvement [34,35,36,37], its higher price and less chemical versatility in surface modification than for hBN restricts its introduction in polymer composites for TC management [38].

The literature indicates that composites of epoxy (EP) resin with cBN or with hBN and cBN have enhanced tribological and anti-corrosion performance, e.g., in water, sea water, and H_2_SO_4_ [39,40,41,42]. This enables use of EP composites with cBN alone or mixed with hBN in applications working in harsh environments, such as geological instrumentation. Considering this idea, polymer composites with cBN might be useful in this field. In particular, EP can be a candidate considering its superior mechanical strength in comparison to other polymers and despite its inherent brittleness, relatively low fracture toughness, and its ability regarding cross-linking and crack initiation. EP is also flammable, releasing a large amount of harmful gases and smoke above 350 °C [43]. On the other hand, EP has excellent insulating properties, and it is frequently used in high-frequency transformers and power electronic equipment such as motors [44]. During operation, epoxy resin ages because of the combined effects of heat, electricity, and mechanical stress, eventually leading to the failure of power equipment [45]. According to various studies, epoxy resin composite materials with high thermal conductivity have an extended lifespan and can prevent overheating and premature aging of equipment [46]. EP composites filled with carbon fibers and h-BN were recently shown to be 3D printable [47].

The aim of this work is to prepare composites based on bicomponent epoxy resin and cubic BN powder filler and to assess, mainly, their thermal conductivity performance. Our results show enhancement of TC by using cBN filler. We also pay attention to other important properties of the composite, such as electrical and mechanical ones.

## 2. Materials and Methods

### 2.1. Materials

**Figure 1:**
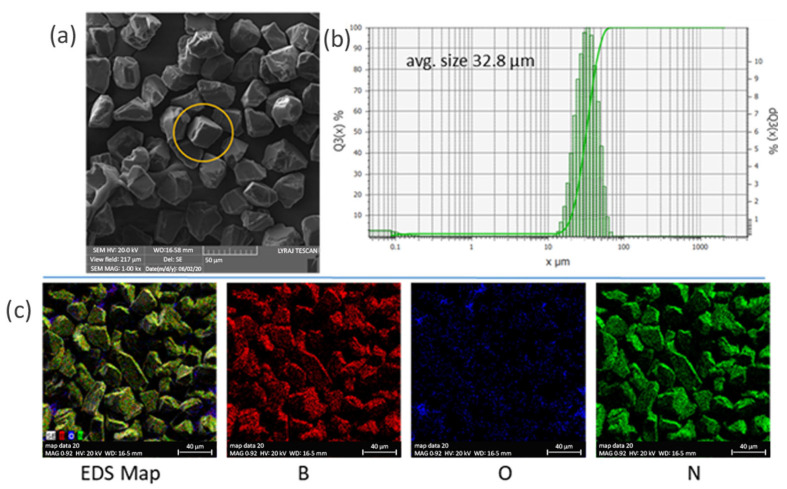
Cubic boron nitride powder: (**a**) SEM image (a cubic particle is shown within a circle); (**b**) granulometric distribution of the powder showing both cumulative and differential fractions: the left Y-axis (Q_3_(X) %) represents the percentage of particles smaller than a given diameter, while the right Y-axis (dQ_3_(X) %) indicates the fraction of particles within specific size ranges; (**c**) EDS elemental maps of B, O, and N.

**Figure 2:**
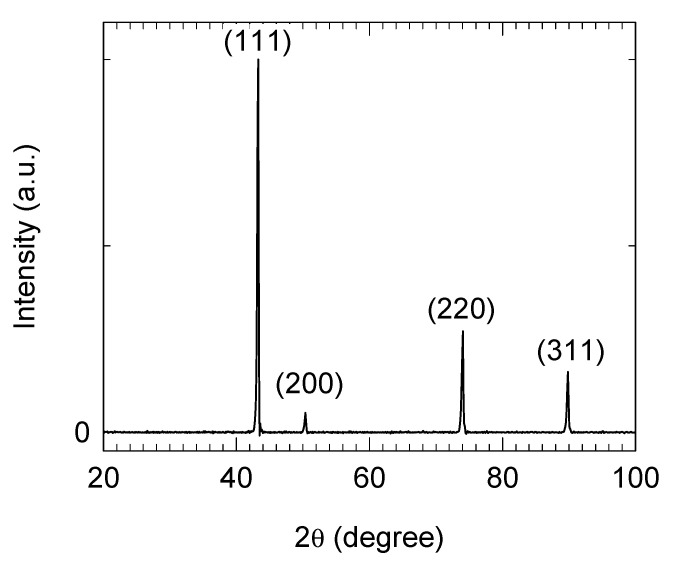
X-ray diffraction pattern of raw cubic boron nitride powder. Phase identification was performed according to the powder diffraction file ICDD 79-0623.

Cubic BN powder was produced by SkySpring NanoMaterials, Inc. (Houston, TX, USA). The morphology of the particles is specific for cubic materials; namely, prisms and cubes can often be visualized (Figure 1a). Edges and corners are sharp like those of hard and brittle materials. The powder granulometric distribution is mono modal with particles between a few microns and 60 µm. The average particle size is 32.8 µm (Figure 1b). EDS maps indicate the presence of oxygen on the surface of the particles (Figure 1c). X-ray diffraction pattern confirms that powder consists of cubic BN phase (Figure 2). No impurities were detected.

The main properties of the commercial bicomponent resin, Elan-tron^®^ MC 4260/W 4260 (ELANTAS EUROPE LLC, Collecchio, Italy), are presented in Table 1. The as-received resin is filled with ceramic fillers of thermal class H (180 °C). This type of EP resin is usually recommended for electric motors, linear motors, and transformers, but expectations are that it can be used successfully in other applications such as geological or other instrumentation working in harsh conditions. It is a resin suitable for large-scale applications, curable at room or moderate temperature, and it has low viscosity, high impregnation properties, and good thermal, electrical, and mechanical properties. The performance parameters provided by the manufacturer are quite similar to those of the epoxy resin WEICON C which, according to Ref. [48], satisfactorily meet the requirements of synchronous motors for electric and hybrid vehicles.

### 2.2. Preparation of Composites

Composite samples of cBN embedded in epoxy resin were prepared from mixtures of epoxy resin and cubic BN powders. The content of cBN was 10, 20, 30, 40, and 50 wt.%. A reference sample without filler (0%) was also prepared (sample ‘1’, Table 2). Two different preparation routes were used: (a) cBN powder (1) was mixed with epoxy base (2) and, afterwards, with hardener (samples ‘2’–‘6’, Table 2); (b) cBN powder (1) was mixed with hardener (3) and, after that, with the epoxy base (2) (samples ‘7’–‘10’, Table 2).

The application of the indicated preparation routes is justified by various possible effects of wetting and dispersion of the filler by the resin’s two components (base and hardener), influencing the polymer polymerization and filler agglomeration in the composite and, thus, modifying the final physical and chemical properties of the product.

**Figure 3:**
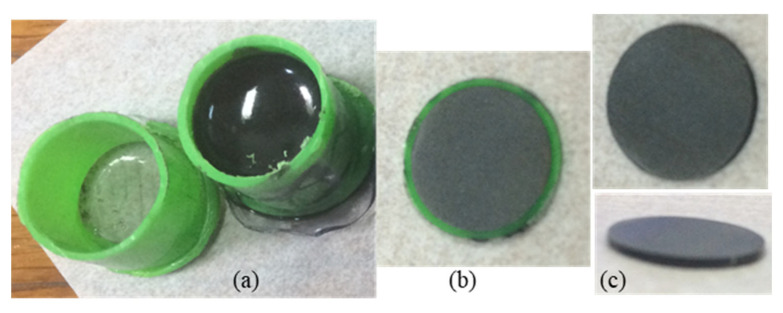
Preparation of composites: (**a**) mold with uncured composite; (**b**,**c**) sample after curing and after being cut into disks of 12.5 mm in diameter and 1 mm thickness for thermal measurements with a Netzsch LFA 457 Microflash system.

The mixing ratio by weight between the base and hardener was constant at 100:10 (or by volume at 100:18) for all samples. The as-obtained mixtures were poured into containers (molds) and cured at room temperature for 48 h (Figure 3a). The mold to obtain samples for thermal and dielectric measurements was manufactured from a PVC pipe with an inner diameter of 12.5 mm and sealed at one end with a PET film at a temperature of 90 °C. For mechanical measurements, a similar procedure was applied using molds from PET with a rectangular shape, resulting in samples of 40 × 4 × 4 mm. After hardening, samples were cut into disks or bars with different thicknesses in order to be characterized by structural, microstructural, thermal, electrical, and mechanical measurements.

### 2.3. Sample Characterization

Bulk density of the samples was measured by Archimedes method in water at room temperature. A Kern ABT 120-5DM balance was used. Results are shown in Table 2.

X-ray diffraction measurements were performed with Bruker-AXS D8 ADVANCE (Karlsruhe, Germany) (Cu_Kα1_ radiation = 1.5406 Å).

Scanning electron microscopy images (Zeiss EVO50, Jena, Germany) were recorded for the raw cBN powder and composite samples.

A Netzsch LFA 457 Microflash system (Selb, Germany) was used to measure thermal diffusivity and specific heat of the composite samples (disks of 12.5 mm in diameter and with 1 mm thickness). Thermal conductivity of the samples was estimated with the formula:

(1) $$\lambda =\alpha \;*\;\rho \;*\;c_{p}$$

where *α* is diffusivity, *ρ* is density, and *c_p_* is specific heat. The density *ρ* of cured samples was measured with Archimedes’ method in water using KERN ALT 220-4M density balance (KERN & SOHN GmbH, Balingen, Germany).

Dielectric spectroscopy (or impedance spectroscopy) measurements were performed by placing samples (discs of 12.5 mm in diameter and 1 mm thickness) between two circular metal electrodes at room temperature. The value of the applied sinusoidal electrical voltage was 100 mV for a frequency range between 0.1 Hz and 10 MHz. An ALPFA-A High Performance Frequency Analyzer, from Novocontrol Technologies GmbH (Montabaur, Germany), was used. Determinations of electrical conductivity, dielectric constant, and dielectric loss tangent at different frequencies were made.

Three-point bending tests at room temperature were carried out with INSTRON 5982 equipment (Norwood, MA, USA). These tests were executed on samples of epoxy composite in the shape of bars with size of 40 mm length, 2 mm width, and 4 mm thickness. Bending load was applied with a speed of 0.2 mm/min.

## 3. Results

### 3.1. XRD of the Composites

**Figure 4:**
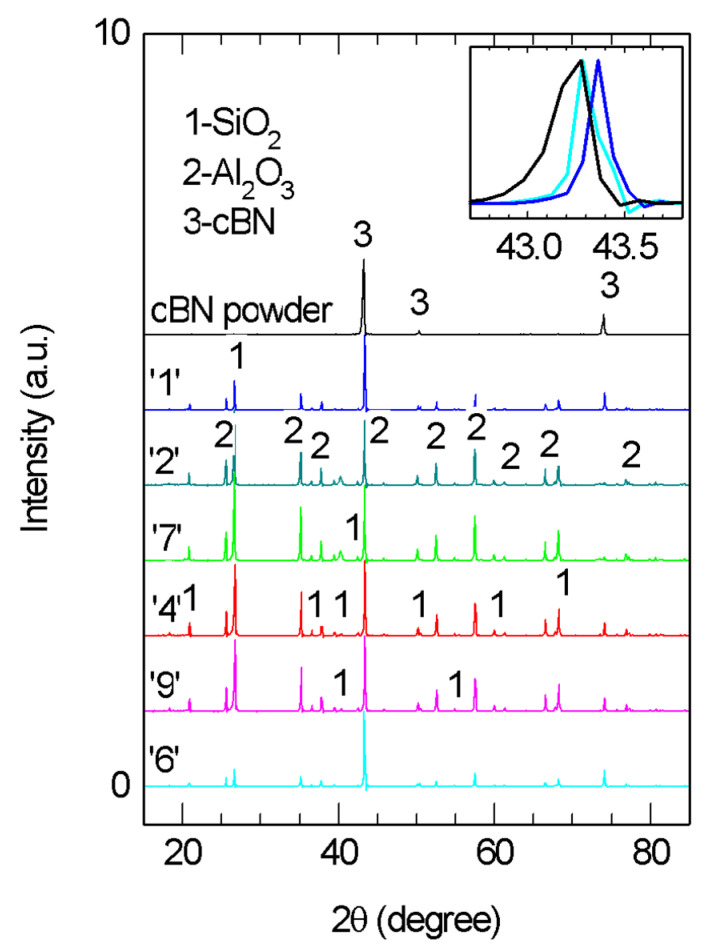
XRD patterns of the cBN raw powder and selected epoxy resin composites ‘1’, ‘2’, ‘7’, ‘4’, ‘9’ and ‘6’. Notations are as in Table 2. Presented spectra are after extraction of the background that includes the influence of the epoxy resin amorphous contribution observed around 2θ–20°.

XRD patterns of selected composite samples and of the raw cBN powder are presented in Figure 4. The most intensive peak in the patterns measured on the composite samples shifts to lower 2θ angles from 43.36 to 43.27° when the amount of cBN additive increases in the composite. As one can observe from the inset to Figure 4, the (111) peak of cBN from the raw powder (Figure 2) is also located at lower 2θ values. This indicates that in the cured epoxy resin with 0 wt.% cBN (sample ‘1’, Table 2), there is another phase, with a peak located at slightly higher 2θ values, overlapping the cBN peak. The second phase was identified as alumina (ICCD 01-083-2080). The manufacturer of the epoxy resin indicates introduction of alumina in the epoxy base, and its role is to increase the thermal conductivity and to improve the flame-retardant property. It is interesting that we also identified another additive in the epoxy resin (sample ‘1’), namely SiO_2_ (ICCD 005-0490), and perhaps it has a role that is similar to that of alumina. According to Refs. [49,50,51], alumina and silica are thermally conductive, with thermal conductivity values of 14–30 W/m·K and 1–14 W/m·K, respectively. These values are significantly lower than for cBN.

No significant differences were observed by XRD (Figure 4) when comparing samples with a constant amount of cBN obtained by different preparation routes (see samples ‘2’ and ‘7’ with 10 wt.% of cBN and ‘4’ and ‘9’ with 30 wt.% of cBN).

### 3.2. SEM Observations and Density of the Composites

**Figure 5:**
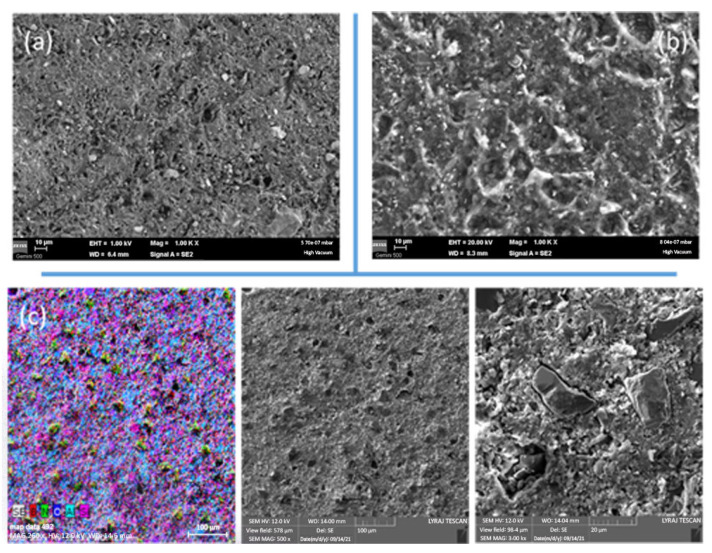
SEM images on the epoxy–cBN composites: (**a**) sample ‘1’ (0% c-BN), (**b**) sample ‘6’ (50% cBN), and (**c**) sample ‘4’ (30% cBN). In (**c**) and RGB, image obtained by overlapping the EDS elemental maps of B (red), N (green), O (blue), Al (light blue), and Si (purple).

Addition of cBN modifies the microstructure (in Figure 5a,b, compare sample ‘1’ with 0 wt.% cBN and ‘6’ with 50 wt.% cBN). The addition of cBN induces coarsening of the microstructure, and roughness increases. The reason for this result is that in the fractured surface, a pull-out mechanism of cBN particles from the resin matrix is active. This can be observed in SEM images taken at different magnifications (Figure 5c), for example, in sample ‘4’ with 30 wt.% cBN. In the cBN-rich samples, due to agglomeration of the filler, the bonding between epoxy resin matrix and cBN is limited, influencing mechanical properties. Details are addressed in Section 3.5.

The presence of Si detected as SiO_2_ by XRD is confirmed by EDS (see EDS RGB map in Figure 5c). In all samples, the EDS concentration ratio between Al and Si was constant at ~2.

No major differences could be revealed by SEM in samples with a constant amount of cBN additive obtained by routes (a) and (b). Nevertheless, samples with a cBN amount equal to or larger than 20 wt.% obtained by route (b) had lower values in density than those fabricated by route (a) (Table 2). This result suggests that route (b) generates lower-quality samples.

### 3.3. Thermal Conductivity of the Composites

**Figure 6:**
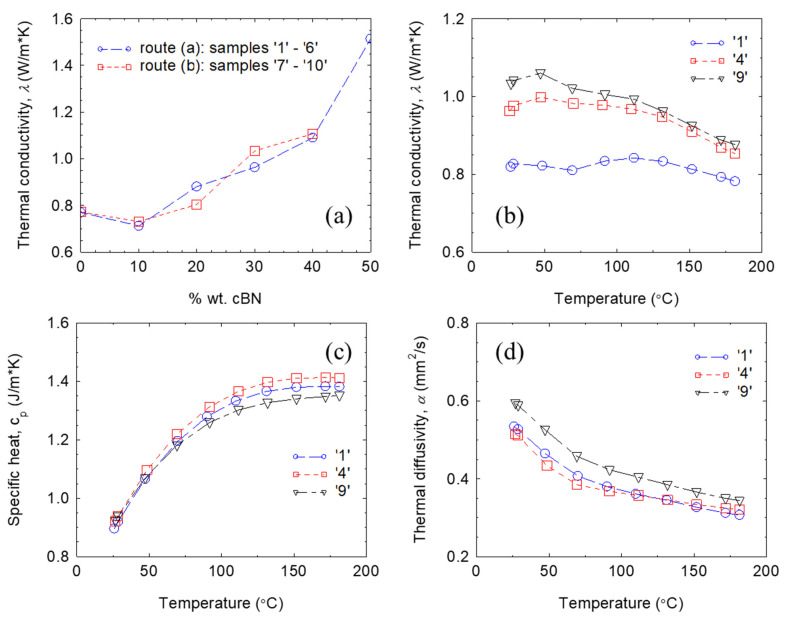
(**a**) Thermal conductivity at room temperature of the composites depending on the c-BN concentration for samples obtained by routes (**a**,**b**); (**b**–**d**) thermal conductivity, specific heat, and diffusivity for selected samples ‘1’ (0 wt.% cBN), ‘4’ (30 wt.% cBN, preparation route (**a**)), and ‘9’ (30 wt.% cBN, preparation route (**b**)).

Room temperature (RT) thermal conductivity *λ* increases in a non-linear manner with the increase of cBN additive (Figure 6a) when the amount of cBN is above 10 wt.%. It attains a maximum value in thermal conductivity of 1.5 W/m·K for the sample with 50 wt.% cBN (sample ‘6’). This *λ*-value is about 1.87 times larger than for sample ‘1’ without the addition of cBN. The trend, apart from some scattering, does not practically depend on the preparation routes. The *λ* increase is relatively slow up to ~30 wt.% cBN, but it is faster at higher cBN concentrations. This can be related to cBN dispersion in the resin matrix and its interaction with the other additives, Al_2_O_3_ and SiO_2_. When the concentration of cBN is less than 30 wt.%, its influence on thermal conductivity is low, and the major role is played by the other additives. Indeed, fillers Al_2_O_3_ and SiO_2_ enhance thermal conductivity of pristine EP from ~ 0.2 W/mK [38] up to 0.77 W/mK in sample ‘1’ without cBN. But, when cBN concentration is higher than 30 wt.%, particles of cBN are not isolated anymore in the composite and can be in direct contact with each other to generate a continuous cBN network where thermal conductivity is high, and it is higher than that of the Al_2_O_3_/SiO_2_ network.

**Figure 7:**
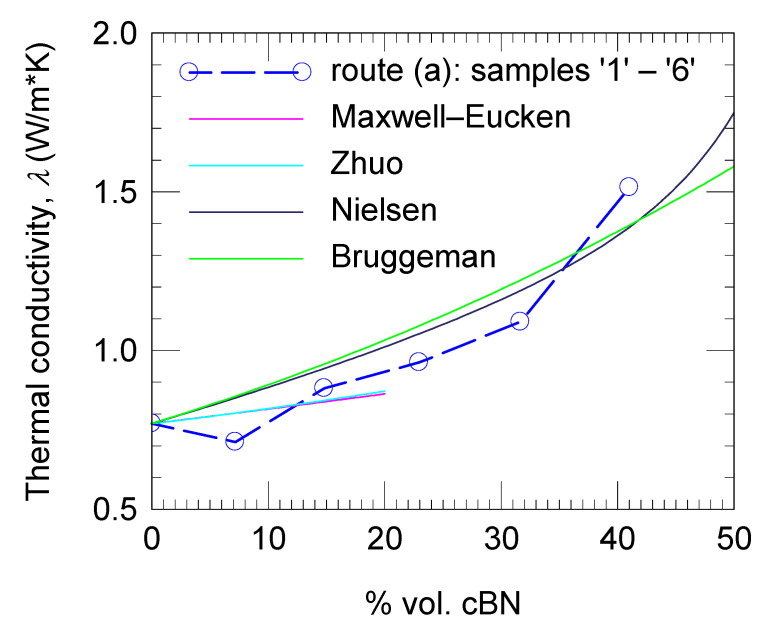
Thermal conductivity vs. volume percent of cBN filler in the composite: experimental data for samples ‘1’–‘6’ fabricated by route (a) and curves obtained by fitting the experimental data with different models (see Table 3).

By applying different conductivity models for composites, namely those of Maxwell–Eucken, Zhuo, Nielsen, and Bruggeman, it was not possible to observe a change in the behavior of the thermal conducting network for an increasing amount of cBN filler in the composite (Figure 7) [52,53,54,55]. Formulas provided by each model and parameters obtained by fitting our experimental data are presented in Table 3. Visually, the Maxwell–Eucken and Zhou models apparently fit well, but the fitting factor r^2^ (0.32 and 0.34) departs significantly from 1, which is the value of r^2^ for excellent fitting. The reason for this is that the applicability domain of the indicated models is restricted to composites with a relatively low filler amount, below 20 vol. %. In our case, in the range 0–20 vol. %, experimental points scatter and are few. In addition, since the amount of Al_2_O_3_ and SiO_2_ is unknown, we arbitrarily applied these models up to 20 vol. % of cBN additive. The Bruggeman and Nielsen models cover the entire compositional range. A better fit is obtained for the Nielsen model (r^2^ = 0.89 is closer to 1 vs. 0.63 for the Bruggeman model), and it can be considered that this model satisfactorily accommodates the experimental data.

Fitting with presented models was performed by fixing thermal conductivity of the matrix at 0.77 W/m·K as it was measured for sample ‘1’ without cBN addition, while the thermal conductivity of the filler *k*_f_ was a free parameter. The parameter *k_f_* extracted by fitting with addressed models uses small values (*k_f_* = 0.85–2.98 W/m·K, see Table 3), namely, at least two orders of magnitude lower than for pristine cBN. This result suggests that the quality of the contacts between the cBN particles is quite poor and influences the percolation features of the filler network, greatly decreasing its thermal conductivity in the composite when compared with pristine cBN. We note that the cBN filler used in this work was in the as-received state. The presence of oxygen on the surface of the cBN particles (Figure 1) and the nature of the conductive network without metallurgical contacts between particles, and possibly with a thin but non-conductive resin layer between them, are among the reasons for the situation encountered. This observation might be more general and applicable to many materials from the same class of TC composites. For example, the composite (12 vol. % BN + 70 vol.% Al_2_O_3_)/PDMS [56] has a thermal conductivity of 3.6 W/mK, the composite with 30 wt.% hBN in EP has a thermal conductivity of 0.82 W/mK [57], the composite with 80 wt. % (Al_2_O_3_-BN) in EP has a thermal conductivity of 1.72 W/mK [58], and the composite with 20 wt.% BNNS covered with flower-like Al_2_O_3_ in cyclic olefin copolymer has a thermal conductivity of ~5.4 W/mK [59], i.e., these values are also low versus those of the fillers. On the other hand, the authors of Ref. [38] prepared epoxy composites with a mixture of hBN and cBN heat-treated at 300 °C for several hours in a vacuum of 10^−2^ torr followed by surface treatment with a silane coupling agent. These composites with clean and surface modified fillers have shown higher thermal conductivities: up to 19 W/mK (26.5 vol. % of filler) [38].

Curves of specific heat *c*_p_ and thermal diffusivity *α* with temperature measured from room temperature and up to 182 °C for samples ‘1’ (without cBN addition), ‘4’ (30 wt.% cBN, preparation route (a)) and ‘9’ (30 wt.% cBN, preparation route (b)) are presented in Figure 6 c and d. Both curves *c*_p_(*T*) and *α*(*T*) show a fast variation at low temperatures (RT − 100 °C); namely, *c_p_* increases and *α* decreases, while at high temperatures (100–182 °C), there is a tendency for saturation. Closer values of *c*_p_ and *α* among mentioned samples are found at RT and at 182 °C, respectively. It is thought that variation in density and microstructural features due to the filler addition and the composite preparation route influence the thermal behavior, hence the variation in *c*_p_ and *α* with temperature. These changes will further impact thermal conductivity *λ*(T) curves (Figure 6b). These curves have a general tendency for a decrease (stronger for added samples ‘4’ and ‘9’) at higher increasing temperatures (T > 100 °C), but shapes are different (the lowest variation with temperature is for unadded sample ‘1’). More research is needed to understand the details. At all temperatures and independent of the preparation route, cBN-added samples ‘4’ and ‘9’ have higher *λ*-values than sample ‘1’ without cBN additive. This result confirms that cBN is a useful additive in practical composites to improve the heat transfer properties.

In summary, results of this section indicate that high concentrations of cBN (over 30 wt.%) are effective for increasing the thermal conductivity in commercial epoxy resins with thermal conductivity that is already enhanced.

### 3.4. Electrical Properties of the Composites

**Figure 8:**
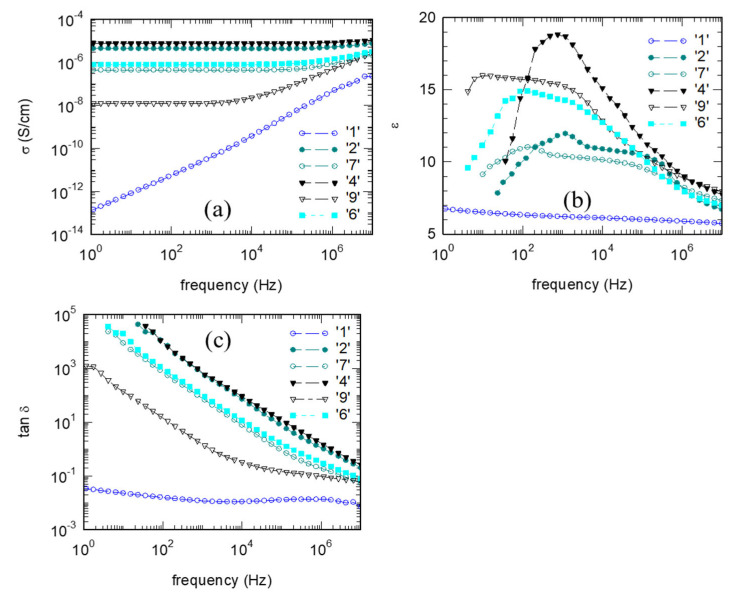
(**a**) Electrical conductivity, (**b**) relative dielectric permittivity (dielectric constant), and (**c**) dielectric loss tangent depending on frequency for selected samples.

The lowest electrical conductivity *σ*, relative permittivity *ε* (dielectric constant), and dielectric loss tangent tan*δ* were measured for the sample ‘1’ without cBN addition. Therefore, electrical insulation is lower, and dissipation is higher in the cBN-added samples than for the cBN-free reference sample. Despite a general worsening in electrical properties with increasing cBN additive concentration in the epoxy resin samples prepared by routes (a) or (b), there is a distinctive pattern (Figure 8a). In samples with intermediate cBN concentrations (e.g., samples ‘2’ and ‘4’ prepared by route (a) and sample ‘7’ prepared by route (b)), conductivity in the entire investigated frequency range is higher than for the reference resin (sample ‘1’) without cBN additive. At higher cBN concentrations, of 30 wt.% or more (e.g., samples ‘6’ prepared by route (a) and ‘9’ prepared by route (b)), there is a partial recovery to a high insulating state. The recovery is stronger for samples prepared by route (b). The pattern observed for *σ* holds relatively well to describe also the behavior of permittivity and loss tangent depending on the c-BN additive concentration (Figure 8b,c). This is appreciated as a quite unusual behavior. In general, in typical composites with one additive, theoretically and experimentally, variation of *σ*, *ε*, or tan*δ* is monotonous with concentration of the filler in the polymer composite, and a recovery of dielectric properties does not occur [29,53].

It is also of interest that the shape of the curves *σ*, *ε*, and tan*δ* with frequency*ν* is not trivial. Typical curves were measured for the reference epoxy resin sample ‘1’ without cBN additive. Conductivity increases with frequency, while *ε* and tan*δ* show a tendency toward a slow decrease that can be considered almost frequency-independent. For the samples with cBN added, the situation changes dramatically. In the conductivity *σ*(*ν*) curves, there is saturation at low frequencies, and this frequency domain is larger when conductivity is higher. The *ε*(*ν*) curves are frequency-dependent and show a more pronounced variation, in the form of a threshold, at higher frequencies, 10^4^–10^5^ Hz. At very high frequencies (10^7^ Hz), *ε* of the composites approaches the values for the cBN-free material. All samples with cBN additive have a dielectric constant two or three times greater than for sample ‘1’ without cBN. Curves of dielectric loss, tan*δ*(*ν*), show a decrease with the increasing frequency, but there is a slowdown (the slope becomes lower) at *ν* > 10^4^ Hz.

The previous two paragraphs indicate a complex dependence of dielectric parameters on the cBN concentration in the composite and on frequency. The conduction mechanisms lead to polarization switching in the direction of the electrical field, resulting in a rapid increase in the load in the area containing the additive. In a polymer, the orientation of the dipoles in the applied electrical field and the relaxation time of the polarization as a function of temperature are accompanied by a switching of the segments in the polymer chain. Thus, the crosslinked structure and interactions between the matrix and filler will determine the dielectric properties of the composite. In our case, the composite is made of four components: epoxy resin, Al_2_O_3_, SiO_2_, and cBN. Also, the influence of residual boron oxide observed by SEM on the surface of cBN raw powder (Section 2.1, Figure 1) should not be neglected. This situation will influence the Maxwell–Wagner–Sillars (MWS) effect, i.e., the interfacial polarization that depends on phases with different dielectric permittivity and conductivity. Additives, their morpho-structural features, their content and distribution in the matrix (clustering), surface state, preparation route, and the interaction between additives and between the additives and the epoxy resin are factors that influence the dielectric properties of the composite. We have introduced some information above showing that there is a pull-out mechanism of cBN particles as evidence for a relatively poor bonding between the cBN and the other components of the composite and that the density of the samples produced by the two routes, (a) and (b), becomes different for higher cBN concentrations. Currently, it is difficult to understand the details of the revealed dependencies due to the overlapping of the complex effects, and more research is needed in this regard.

### 3.5. Mechanical Properties of the Composites

**Figure 9:**
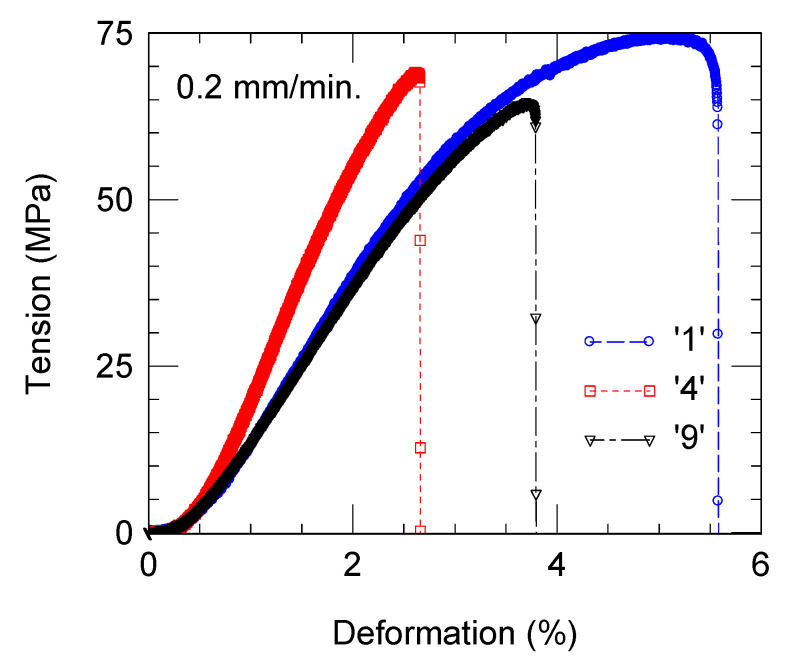
Bending strength vs. strain for samples ‘1’, ‘4’, and ‘9’ (sample notation is as in Table 2). Photos (on the right side) present fractured composite samples after bending test.

For selected composites, strength–strain bending curves are presented in Figure 9. The addition of cBN decreases mechanical properties. Bending strength and strain until fracturing are lower in samples containing cBN. Comparative to the reference epoxy resin without cBN, a stronger decrease is encountered for the strain, from 5.58% in the cBN-free sample to 2.65% or 3.78% in the samples with 30 wt.% cBN fabricated by route (a) and (b), respectively. At the same time, bending strength in these samples decreases from 74 to 70 or 64 MPa.

The strength–strain curve for sample ‘4’ with 30 wt.% cBN prepared by route (a) has a shape closer to that of the reference cBN-free sample ‘1’ than for sample ‘9’ with 30 wt.% cBN prepared by route (b). Sample ‘9’ has a higher elasticity module and relatively high strength. This sample has a lower strain, indicating a stronger brittle behavior.

One observes that as for electrical properties, mechanical properties are sensitive to the preparation route of the composite. To some extent, this is related to cBN particles’ pull-out mechanism, which may depend on the preparation route (a) or (b). For example, the preparation route of the composite promoted some differences in the sample’s density; however, small and unrevealed differences in the microstructural details may also contribute to composite fracture.

## 4. Conclusions

Composites with a resin matrix containing Al_2_O_3_ and SiO_2_ and a cBN filler (0–50 wt.%) were prepared and investigated. Two preparation routes were used. In the first one (a), cBN powder was first mixed with epoxy resin base and, after that, with the hardener. In the second one (b), cBN powder was first mixed with hardener and, afterwards, with the resin base. Regardless of the preparation routes, an enhancement in the thermal conductivity, from 0.77 W/m·K in the reference resin without the cBN additive to 1 and 1.5 W/m·K in the composites filled with 30 and 50 wt.% cBN, respectively, was measured. On the other hand, there is a reasonably low decrease in bending strength from 74 for 0 wt.% cBN to 70 or 64 MPa for 30 wt.% cBN added by method (a) or (b). Strain at breakage also decreased from 5.58% to 2.65 or 3.78%, indicating a more brittle behavior. Dielectric properties measured for frequencies of 1–10^7^ Hz were sensitive depending on cBN concentration so that the shape of the curves of the dielectric parameters, such as electrical conductivity *σ*, relative permittivity *ε* (dielectric constant), and dielectric loss tangent tan*δ* vs. frequency, were modified. The general tendency was to decrease the insulating characteristics. But in samples with 30 wt.% or more of cBN, a partial recovery to a high insulating state was observed. The sample with 30% wt. cBN has a balanced package of functional properties, which is potentially of interest for electrotechnical and geological applications. Thermal properties were almost not sensitive to the processing routes, but mechanical and dielectric properties were dependent on them.

## Figures and Tables

**Table 1:** Epoxy resin properties in the liquid and cured state. Tg is temperature of glass transition.

**Resin in the liquid state:**	
Viscosity of the mixed (base and hardener) components	2500−5500 mPas (20 °C)
Mixing ratio by weight	100:10
Suggested curing cycles	48 h at 25 °C or 6 h at 80 °C
**Resin in the cured state 24 h at RT + 15 h at 60 °C**	
Glass transition temperature	55−65 °C
Thermal conductivity (room temperature, RT = 23 ± 2 °C)	~0.7 W/m·K
Electrical volume resistivity (RT)	>10^13^ Ω·cm
Linear thermal coefficient of expansion	60–70 × 10^−6^ °C (Tg − 10 °C) 135–155 × 10^−6^ °C (Tg +10 °C)
Dielectric constant (RT)	3.5−4.5
Loss factor (RT)	10−30 × 10^−3^
Bending strength	75−85 MPa

**Table 2:** cBN concentration, mixture density, and preparation route.

Sample	cBN Content (wt.%) (1)	Mixture Density (g/cm^3^)	Epoxy Base (2) + Hardener (3) (wt.%)	Preparation Route
‘1’	0	1.745	100	(2) + (3)
‘2’	10	1.845	90	method (a): [(1) + (2)] + (3)
‘3’	20	1.929	80	
‘4’	30	2.013	70	
‘5’	40	2.113	60	
‘6’	50	2.224	50	
‘7’	10	1.825	90	method (b): [(1) + (3)] + (2)
‘8’	20	1.884	80	
‘9’	30	1.885	70	
‘10’	40	2.00	60	

**Table 3:** Models of thermal conductivity in composites and parameters obtained from fitting experimental data for samples ‘1’–‘6’ fabricated by route (a).

Model	Formula	*k_f_*	Fitting Factor (r^2^)
Maxwell–Eucken	$k_{c}=k_{m}\frac{2k_{m}+k_{f}+2V_{f}(k_{f}-k_{m})}{2k_{m}+k_{f}-V_{f}(k_{f}-k_{m})}$	0.856	0.32
Zhuo	$k_{c}=k_{m}\left[1+\frac{V_{f}(k_{f}-k_{m})}{k_{f}-V_{f}^{\frac{1}{3}}\left(k_{f}-k_{m}\right)}\right]$	1.47	0.34
Nielsen	$k_{c}=\frac{1+ABV_{f}}{1-B\psi V_{f}};\;\;B=\frac{\frac{k_{f}}{k_{m}}-1}{\frac{k_{f}}{k_{m}}+A};\;\;\psi =1+\frac{V_{f}^{2}\left(1-V_{m}\right)}{V_{m}^{2}};$ A=2 for cubic shape [54]	2.82	0.89
Bruggeman	$1-V_{f}=\frac{k_{f}-k_{c}}{k_{f}-k_{c}}{\left(\frac{k_{m}}{k_{c}}\right)}^{\frac{1}{3}}$	2.98	0.63

Note: *k_c_*, *k_m_*, and *k_f_* are the specific thermal conductivities of the composite, of the epoxy resin (*k_f_* = 0.77 W/m·K), and of the filler; *V_m_*, and *V_f_* are volume fractions of the matrix and filler in the composite.

## Data Availability

The original contributions presented in this study are included in the article. Further inquiries can be directed to the corresponding authors.
